# Peripheral Nerve Injury in War Surgery

**Published:** 1919

**Authors:** 


**Peripheral Nerve Injury in War Surgery. Routes of Approach.** By Major C. S. VENABLE, M. C., Base Hospital No. 41, A. E. F. Abstract of an article to be published in the *Southern Medical Journal.*

*Upper Extremities.*

Injuries at the root of organization of the cervical segments going to make up the brachial plexus may be distinguished from those involving the cords after organization by the escape of the thoracic and scapular branches, grossly excluding paralysis of the pectoralis major and minor, serratus magnus and scapular muscles. Involvement of certain segments may be localized according to their ultimate distribution in the event the whole is not destroyed. If the patient is imagined to be stooping at 90.0, the fingers pointing straight down and the thumb pointing outwards, the surgical approach is through an angulated incision from below the mastoid tip downward along the posterior of the sterno-mastoid to the inner third of the clavicle, thence outward along its inferior border to its end, the clavicle being divided at the junction of the inner and middle third, and the flap reflected outward; the plexus appears coming from behind the scalenus anticus, traversing the scalenus medius at a point opposite one finger's breadth below the thyroid cartilage and behind the outer border of the sterno-mastoid muscle.

*The long thoracic nerve* arises from the cervical plexus before organization of the brachial plexus. The surgical approach is the same as that for the brachial plexus.

*The external anterior thoracic* arises from the cervical plexus before organization of the brachial plexus. The resulting loss of function is arm adduction and a flabby anterior axillary fold. The surgical approach is that given for the brachial plexus.

*The supra-scapular.* Here the loss of function is a weakened inward and outward rotation and abduction of the humerus.

*The musculo-cutaneous* arises from the anterior cord of the plexus. The impairment of function is a weakened flexion of the arm on the shoulder and of the forearm on the arm. The surgical approach is through the fascial plane between the coraco-brachialis and the short head of the biceps.

*The ulna* arises from the inferior cord of the brachial plexus. The loss of function as a result of injury above the elbow is absence of adduction of the wrist with the hand in supernation, flexion of the distal phalanges of the 4th and 5th fingers, abduction and adduction of the fingers, and adduction of the thumb.

The surgical approach in the arm is along the course of the brachial vessels, the nerve lying to the inner side of the median. In the forearm it lies in the fascial plane just beneath the flexor carpi ulnaris.

*The median* arises from the inferior cord of the brachial plexus, resulting in sensory and motor disturbances. Clinically there is loss of pronation of the forearm, flexion of the wrist, flexion of the thumb and fingers, except the last phalanges of the fourth and fifth, and abduction and transpalmar adduction of the thumb. The surgical approach in the arm is along the course of the brachial artery, in the forearm, in the fascial plane beneath and to the ulna side of the flexor carpi radialis.

*The musculo-spiral* arises from the posterior cord of the brachial plexus. Injury to this nerve results in paralysis and sensory troubles. The impairment of function is weakened extension of the forearm on the wrist, extension of the thumb and the proximal phalanges of all the fingers. The surgical approach to the first portion is that outlined for the brachial plexus; the second portion, the axillary route, and in the mid-arm at the site of the insertion of the deltoid.

### *Lower Extremities.*

The lumbar plexus arises from the first to the 4th lumbar nerves, each dividing into upper and lower branches, supplying sensation to the genitals and scrotum, to the skin of the upper one-fourth of the front of the thigh, and to that of the outer side and back of the thigh.

*The anterior crural* arises from filaments from the first and second segments of the lumbar plexus, furnishing motor supply to the quadriceps femoris, sartorius and pectoneus, and sensation to the thigh and its lower 3/4.

*The obturator* arises from the lower branches of the 3rd and 4th segments and supplies the adductors of the thigh. Motor paralysis following injury involving the anterior crural and obturator produces the following disturbances of function : loss of thigh flexion, leg extension and leg adduction. The surgical approach is above Poupart's ligament through an incision one inch below the crest of the ilium, reflecting the flap over the crest sub-periostially and proceeding sub-periostially across the crest of the iliac fossa. The approach to the anterior crural below Poupart's ligament is through an incision along the border of the sartorius reflecting the muscle outwards.

*The sacral plexus* supplies the semi-membranosis, semi-tendonosis biceps and [illegible]dductor magnus, in part. Functional disturbances resulting would be weakened knee flexion, as the only knee flexors remaining are the gracilis, sartorius and gastrocnemious. The surgical approach is through an incision in the long axis of the thigh.

*The external popliteal* crosses obliquely from a point 4 to 6 inches above the knee along the inner side of the biceps to the inner side of the head of the fibula. The loss of function comprises loss of dorsiflexion of the foot with adduction, extension of the great toe, and extension of the lesser toes with adduction of the foot. The surgical approach to the external popliteal is through an incision at the inner side of the fibula.

*The internal popliteal* is a continuation of the great sciatic. Functional disturbances following injury are weakened knee flexion, loss of plantar flexion of the foot, and paralysis of muscles supplied by the posterior tibial. Surgical approach is through the popliteal space in its long axis.

The posterior tibial is the continuation of the internal popliteal from the low border of the popliteus muscle. The functional disturbances resulting from injury are weakened plantar flexion of the foot, loss of adduction with pronation, and adduction and abduction of the toes. Surgical approach in its upper portion is through the popliteal space and traced downwards.

